# A phenol amine molecule from *Salinivenus iranica* acts as the inhibitor of cancer stem cells in breast cancer cell lines

**DOI:** 10.1038/s41598-023-39736-9

**Published:** 2023-08-04

**Authors:** Atefeh Safarpour, Marzieh Ebrahimi, Seyed Abolhassan Shahzadeh Fazeli, Mohammad Ali Amoozegar

**Affiliations:** 1 Extremophiles Laboratory, Department of Microbiology, School of Biology and Center of Excellence in Phylogeny of Living Organisms, College of Science, University of Tehran, Tehran, Iran; 2 Department of Stem Cells and Developmental Biology, Cell Science Research Center, Royan Institute for Stem Cell Biology and Technology, ACECR, Tehran, 19395-4644 Iran; 3 Department of Molecular and Cellular Biology, Faculty of Basic Sciences and Advanced Technologies in Biology, University of Science and Culture, ACECR, Tehran, Iran

**Keywords:** Biotechnology, Oncology

## Abstract

In recent years, the anticancer properties of metabolites from halophilic microorganisms have received a lot of attention. Twenty-nine halophilic bacterial strains were selected from a culture collection to test the effects of their supernatant metabolites on stem cell-like properties of six human cancer cell lines. Human fibroblasts were used as normal control. Sphere and colony formation assay were done to assess the stem cell-like properties. invasion and migration assay, and tumor development in mice model were done to assess the anti-tumorigenesis effect in vitro and in vivo. The metabolites from *Salinivenus iranica* demonstrated the most potent cytotoxic effect on breast cancer cell lines (IC50 = 100 µg/mL) among all strains, with no effect on normal cells. In MDA-MB-231 cells, the supernatant metabolites enhanced both early and late apoptosis (approximately 9.5% and 48.8%, respectively) and decreased the sphere and colony formation ability of breast cancer cells. Furthermore, after intratumor injection of metabolites, tumors developed in the mice models reduced dramatically, associated with increased pro-apoptotic caspase-3 expression. The purified cytotoxic molecule, a phenol amine with a molecular weight of 1961.73 Dalton (IC50 = 1 µg/mL), downregulated pluripotency gene SRY-Box Transcription Factor 2 (*SOX*-2) expression in breast cancer cells which is associated with resistance to conventional anticancer treatment. In conclusion, we suggested that the phenol amine molecule from *Salinivenus iranica* could be a potential anti-breast cancer component.

## Introduction

Cancer stem cells are small populations in tumor cells responsible for cancer complications like drug resistance and metastasis^1–3^. Cancer stem cell targeting is an intelligent approach for increasing the survival rate in cancer treatment^4,5^. Several investigations have screened natural products for their inhibitory effects on cancer stem cells^6^. Among these natural products, Salinomycin, a metabolite generated from the soil bacterium *Streptomyces albus*, is one of the natural products that directly kills and inhibits cancer stem cells and is on the verge of being approved by the Food and Drug Administration (FDA) as an effective anti-cancer drug with few side effects^7^. This evidence supports the idea that microorganisms can be used in cancer stem cell targeting.

Halophiles are a group of extremophile microorganisms that live in high salt concentrations^8^. Recent scientific research has emphasized the significance of metabolites derived from halophilic microorganisms for treating cancer. Specifically, two metabolites known as 8-O-Methyltetrangulol and Naphthomycin A were isolated from the halophilic strain *Streptomyces* sp. WH26 and have shown promising cytotoxic activity against various types of cancer cell lines. These include human lung cancer cells (A549), human cervical cancer cells (HeLa), human hepatocellular carcinoma cells (BEL-7402), and human colorectal adenocarcinoma cells (HT-29)^9^. In another study, researchers found that five of forty moderately halophilic bacterial strains reduced the viability of human hepatocellular carcinoma Bel 7402 cells to 50% when tested at 40 µg/mL^10^. In 2019 Neelam et al., reported that metabolites from *Piscibacillus* sp. C12A1 isolated from Sambhar Lake in India decreased the viability of the MDA-MB-231 breast cancer cell line with downregulation of *Bcl*-xL and *CDK*-2 expression in these cells. Also, these metabolites inhibited cell migration and colony formation of MDA-MB-231 cancer cells^11^.

Despite the abovementioned evidence, no research has been done on the effect of metabolites from halophiles or extremophiles on cancer stem cells; therefore, studies in this regard are necessary. This study screened the cytotoxic effect of supernatant metabolites (SM) from twenty-nine native halophilic and halotolerant strains obtained from the Iranian biological resource center (IBRC) on six distinct cancer cell lines, including lung (A549), prostate (DU145, LNCaP, PC3), breast (MCF-7, MDA-MB-468), and human fibroblasts as the normal control. It is important to note that the cancer cell lines used in this study are heterogeneous and include only a small subset of stem cells. The mechanism of action and tumorigenicity of strains that selectively reduced viability only on cancer cells and stem cell-like properties of cancer cells and not normal cells were assessed through in vitro and mouse models. In vitro assays, such as colony formation and sphere formation, were used to demonstrate the stem cell-like characteristics of these cancer cells. As the selected SMs showed potential anticancer effects on breast cancer cells, the MDA-MB-231 breast cancer cell line was added to the study and used alongside the other two breast cancer cell lines. Furthermore, the effector molecule of the supernatant metabolite was purified, and its structure was partially elucidated.

## Results

### Screening and finding the most cytotoxic strains against different cancer lines

Twenty-nine halophilic and halotolerant bacterial strains were obtained from the Iranian biological resource center (IBRC) microorganisms bank, all of which were type strains of the species (Table 1). To screen the anti-cancer effect of the selected strains, three cancer types were chosen; prostate cancer (DU145, LNCaP, PC3), breast cancer (MDA-MB-468, MCF-7), and lung carcinoma (A549). Human fibroblasts (HFF-5) were used as normal cells. All cells were treated with different concentrations of Supernatant Metabolites (SM) of those selected strains. As shown in Table 1, the SM of eleven strains like *Alloactinosynnema iranicum*, *Alteribacillus persepolensis*, *Bacillus iranensis*, *Bacillus persicus*, *Cyclobacterium halophilum*, *Limimonas halophila*, *Oceanobacillus limi*, *Pseudomonas salegens*, *Salinicoccus iranensis*, *Salinivibrio proteolyticus*, and *Thalassobacillus cyri* did not have any cytotoxic effect on fibroblasts and cancer cells.

**Table 1:** Effect of SM of halophilic and halotolerant bacterial strains on the viability of normal and tumor cells.

Cell lines strain	HFF-5	PC3	LNCaP	DU145	A549	MDA-MB-468	MCF-7	MDA-MB-231
*Salinivenus iranica*	**800 µg/mL**	**400 µg/mL**	**200 µg/mL**	**400 µg/mL**	**400 µg/mL**	**100 µg/mL**	**100 µg/mL**	**100 µg/mL**
IBRC M 10036	36.75 ± 1.39	55.87 ± 0.48	52.62 ± 2.51	57.9 ± 0.41	58.92 ± 1.05	50.71 ± 2.32	50.97 ± 3.24	50.97 ± 3.24
MGM 23%								
*Salinibacter ruber*	**400 µg/mL**	**200 µg/mL**	**100 µg/mL**	**200 µg/mL**	**200 µg/mL**	**100 µg/mL**	**100 µg/mL**	**100 µg/mL**
IBRC M 10423	51.85 ± 2.54	59.22 ± 2.55	50.49 ± 2.45	45.72 ± 4.12	55.83 ± 2.54	53.40 ± 2.42	58.14 ± 1.52	50.97 ± 3.24
MGM 23%								
*Salinivenus lutea*	**200 µg/mL**	**400 µg/mL**	**200 µg/mL**	**800 µg/m**	**400 µg/mL**	**200 µg/mL**	**400 µg/mL**	NT
IBRC M 10423	50.15 ± 0.81	55.27 ± 5/01	51.80 ± 1.14	42.68 ± 1.51	58.04 ± 3.31	44.14 ± 2.00	34.35 ± 2.70	
MGM 23%								
*Aquibacillus albus*	**800 µg/mL**	**800 µg/mL**	**800 µg/mL**	**800 µg/mL**	**800 µg/mL**	**200 µg/mL**	**400 µg/mL**	NT
IBRC M 10798	17.06 ± 3.68	16.07 ± 0.13	33.87 ± 3.03	19.42 ± 2.08	53.01 ± 4.46	28.72 ± 0.13	23.84 ± 1.79	
SWN 7%								
*Aliicoccus persicus*	**800 µg/mL**	**800 µg/mL**	**800 µg/mL**	**800 µg/mL**	**800 µg/mL**	**200 µg/mL**	**800 µg/mL**	NT
IBRC M 10081	59.9 ± 6.95	27.46 ± 6.78	36.02 ± 1.51	33.19 ± 1.22	50.81 ± 4.10	50.36 ± 4.97	21.38 ± 1.23	
SWN 7%								
*Marinobacter persicus*	**800 µg/mL**	**800 µg/mL**	**800 µg/mL**	**800 µg/mL**	–	**200 µg/mL**	**400 µg/mL**	NT
IBRC M 10445	49.09 ± 4.49	38.80 ± 4.59	53.06 ± 2.54	50.56 ± 0.20		41.74 ± 5.24	45.87 ± 1.48	
SWN 3%								
*Ornithinibacillus halophilus*	–	**800 µg/mL**	**200 µg/mL**	**800 µg/mL**	–	**400 µg/mL**	**400 µg/mL**	NT
IBRC M 10683		13.76 ± 0.20	44.42 ± 2.73	46.60 ± 1.40		40.00 ± 1.30	47.27 ± 0.43	
SWN 3%								
*Bacillus halosaccharovorans*	–	–	**800 µg/mL**	–	–	**400 µg/mL**	**800 µg/mL**	NT
IBRC M 10095			44.90 ± 1.34			50.31 ± 0.12	45.02 ± 2.29	
SWN 3%								
*Alteribacillus bidgolensis*	–	–	–	–	–	**400 µg/mL**	**800 µg/mL**	NT
IBRC M 10614						48.30 ± 4.27	40.12 ± 3.73	
SWN 7%								
*Salinithrix halophila*	–	**800 µg/mL**	**800 µg/mL**	–	–	–	–	NT
IBRC M 10813		49.45 ± 1.50	46.72 ± 3.71					
SWN 3%								
*Bacillus salsus*	–	–	–	–	**100 µg/mL**	–	–	NT
IBRC M 10078					53.97 ± 11.4			
SWN 3%								
*Aquibacillus halophilus*	–	–	–	–	–	**800 µg/mL**	–	NT
IBRC M 10775						26.58 ± 0.48		
SWN 7%								
*Aquibacillus korensis*	–	–	–	–	–	**800 µg/mL**	–	NT
IBRC M 10657						31.12 ± 1.08		
SWN 7%								
*Halobacillus karajensis*	–	–	–	–	–	**800 µg/mL**	–	NT
IBRC M 10221						39.20 ± 0.23		
SWN 3%								
*Lentibacillus persicus*	–	–	–	–	–	**400 µg/mL**	–	NT
IBRC M 10440						51.20 ± 0.19		
SWN 3%								
*Piscibacillus halophilus*	–	–	–	–	–	**800 µg/mL**	–	NT
IBRC M 10220						45.46 ± 2.83		
SWN 7%								
*Saliterribacillus persicus*	–	–	–	–	–	**800 µg/mL**	–	NT
IBRC M 10629						37.90 ± 2.42		
SWN 3%								
*Salinispirillum marinum*	–	–	**800 µg/mL**	–	–	–	–	NT
IBRC M 10765			38.79 ± 0.85					
SWN 3%								
*Alloactinosynnema iranicum*	–	–	–	–	–	–	–	NT
IBRC M 10403								
ISPII 0.5%								
*Alteribacillus persepolensis*	–	–	–	–	–	–	–	NT
IBRC M 10436								
SWN 7%								
*Bacillus iranensis*	–	–	–	–	–	–	–	NT
IBRC M 10446								
SWN 3%								
*Bacillus persicus*	**–**	**–**	**–**	**–**	**–**	**–**	**–**	NT
IBRC M 10115								
SWN 3%								
*Cyclobacterium halophilum*	**–**	**–**	**–**	**–**	**–**	**–**	**–**	NT
IBRC M 10761								
SWN 3%								
*Limimonas halophila*	**–**	**–**	**–**	**–**	**–**	**–**	**–**	NT
IBRC M 10018								
MGM 23%								
*Oceanobacillus limi*	**–**	**–**	**–**	**–**	**–**	**–**	**–**	NT
IBRC M 10780								
SWN 3%								
*Pseudomonas salegens*	**–**	**–**	**–**	**–**	**–**	**–**	**–**	NT
IBRC M 10762								
SWN 3%								
*Salinicoccus iranensis*	**–**	**–**	**–**	**–**	**–**	**–**	**–**	NT
IBRC M 10198								
SWN 7%								
*Salinivibrio proteolyticus*	**–**	**–**	**–**	**–**	**–**	**–**	**–**	NT
IBRC M 10218								
SWN 3%								
*Thalassobacillus cyri*	**–**	**–**	**–**	**–**	**–**	**–**	**–**	NT
IBRC M 10743								
SWN 3%								

The numbers are referred as ≤ IC50. -: The SM did not reduce the viability of cells to ~ 50% in any of examined concentrations. Fibroblast (HFF-5), prostate cancer (PC3, LNCaP, and DU145), lung cancer (A549), and breast cancer (MDA-MB-468, MDA-MB-231 and MCF-7) cell lines. *SWN* Sea Water Nutrient Medium, *MGM* Modified Growth Medium, *ISPII* Yeast Malt Medium, *NT* Not Tested.

Significant values are in bold.

**Figure 1:**
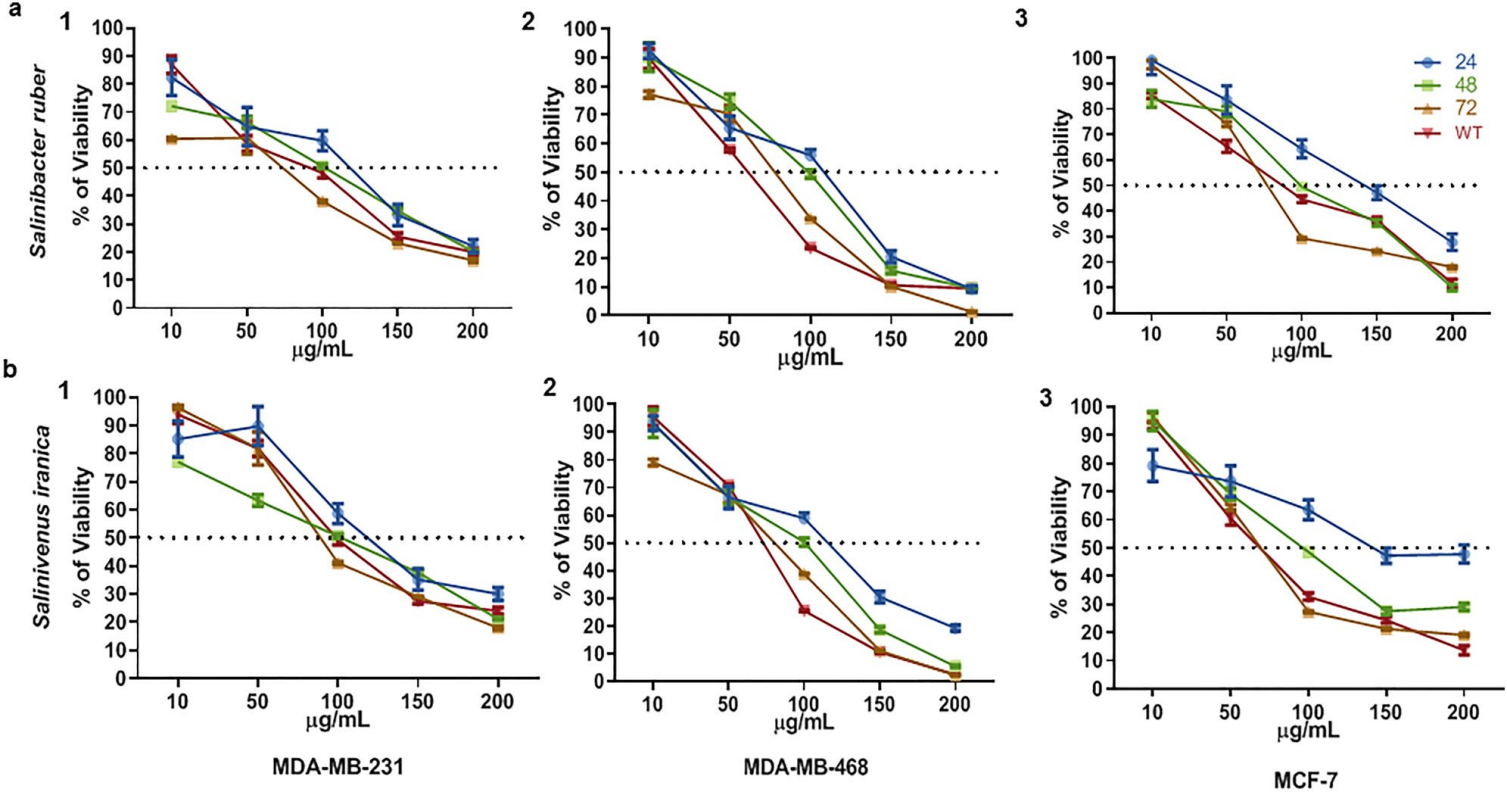
Finding the IC50 dose of *Salinibacter ruber* and *Salinivenus iranica* on breast cancer cell lines by MTT assay. (**a**) Effect of 10, 50, 100, 150 and 200 µg/mL of *Salinibacter ruber* metabolites in 24, 48, 72 h and after 48 h treatment and 48 h washout (WT) on 1. MDA-MB-231, 2. MDA-MB-468, and 3. MCF-7 cells. The viability of cells in 48 h and WT groups reduced to 50% after treatment with 100 µg/mL dose. (**b**) Effect of 10, 50, 100, 150 and 200 µg/mL of *Salinivenus iranica* metabolites in 24, 48, 72 h and after 48 h treatment and 48 h washout (WT) on 1. MDA-MB-231, 2. MDA-MB-468, and 3. MCF-7 cells. The viability of cells in 48 and WT groups was reduced to 50% after treatment with 100 µg/mL dose. n = 3.

However, *Salinivenus lutea* was cytotoxic on normal fibroblasts even at a 200 µg/mL dose. *Aliicoccus persicus* and *Aquibacillus albus* did not have any effect at a 100 µg/mL dose on cell viability in all cells. Meanwhile, they were heavily toxic for both normal fibroblast and cancer cells at 400 µg/mL and 800 µg/mL. Twelve strains were not toxic for fibroblasts and only affected cell viability of one or two tumor types but at 400 µg/mL and 800 µg/mL. Among twenty-nine selected strains, we found *Salinibacter ruber* and *Salinivenus iranica* with an anti-cancer effect on all breast cancer cell lines (MCF7, MDA-MB-231, MDA-MB-468) at 100 µg/mL and without any effect on normal fibroblasts (Table 1). The SM of both strains significantly reduced the viability of cells to 50% 48h post-treatment, and their effect was stable for an extra 48h when the SM was removed from culture media (Fig. 1a,b). Hence, these strains were selected for further experiments.

### *Salinivenus iranica*, *Salinibacter ruber*, and the cancer stem cell-like properties of breast cancer cell lines

**Figure 2:**
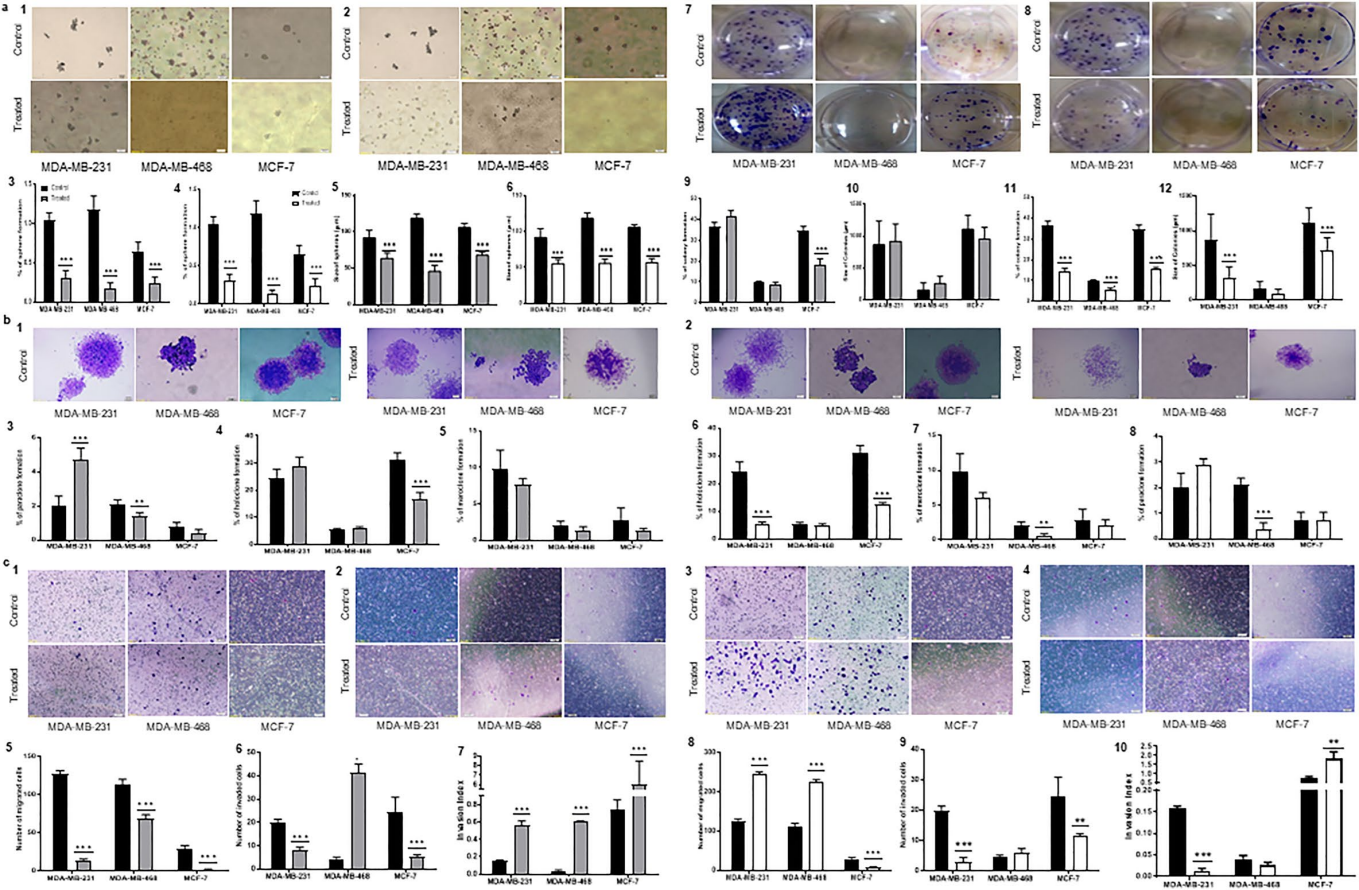
Effect of *S. ruber* and *S. iranica* SM (100 µg/mL) on cancer stem cell properties of breast cancer cell lines after 48h treatment. (**a**) Sphere formation. 1. Sphere formation in *S. ruber*. 2. Sphere formation in *S. iranica*. 3. Percentage of sphere formation in *S. ruber*. 4. Percentage of sphere formation in *S. iranica*. 5. Size of spheres in *S. ruber*. 6. Size of spheres in *S. iranica*. (**b**) Colony formation. 1. Colony formation in *S. ruber*. 2. Colony formation in *S. iranica*. 3. Percentage of colony formation in *S. ruber*. 4. Size of colonies in *S. ruber*. 5. Percentage of colony formation in *S. iranica*. 6. Size of colonies in *S. iranica*. 7. The shape of colonies in *S. ruber*. 8. The shape of colonies in *S. iranica*. 9. Percentage of holoclones in colonies in *S. ruber*. 10. Percentage of meroclones in colonies in *S. ruber*. 11. Percentage of paraclones in colonies in *S. ruber*. 12. Percentage of holoclones in colonies in *S. iranica*. 13. Percentage of meroclones in colonies in *S. iranica*. 14. Percentage of paraclones in colonies in *S. iranica*. (**c**) Migration and invasion. 1. Migration test in *S. ruber*. 2. Invasion test in *S. ruber*. 3. Migration test in *S. iranica*. 4. Invasion test in *S. iranica*. 5. Migration number of Breast cancer cell lines in *S. ruber*. 6. Invasion number of Breast cancer cell lines in *S. ruber*. 7. Invasion index of Breast cancer cell lines in *S. ruber*. 8. Migration number of Breast cancer cell lines in *S. iranica*. 9. Invasion number of Breast cancer cell lines in *S. iranica*. 10. Invasion index of Breast cancer cell lines in *S. iranica*. n = 4, **p < 0.01, ***p < 0.001.

Sphere and colony formation determine the self-renewal potential of cancer cells^12,13^. Therefore, we evaluated the effect of *Salinibacter ruber* and *Salinivenus iranica* SMs on sphere and colony formation ability of MDA-MB-231, MDA-MB-468, and MCF-7 cells. Prior to treatment, all three human breast cancer cell lines exhibited stem-like properties, as evidenced by their ability to form colonies and large spheres. These characteristics are indicative of the presence of stem-like cells in the cell lines. Our results indicated that cells in all treated groups with both SMs lost their repopulation and could not form large spheres. *Salinivenus iranica* and *Salinibacter ruber* reduced the spheroid formation ability to about 3.5, 8.4, and 2.7 folds in MDA-MB-231, MDA-MB-468, and MCF-7 cells, respectively (*p* < 0.001). In addition, *Salinivenus iranica* and *Salinibacter ruber* SMs significantly decreased the size of spheres about 1.4-fold in MDA-MB-231 cells, 2.2 folds in MDA-MB-468, and 1.5-fold in MCF-7 cells (Fig. 2a,1–6). The results of the colony formation test were a bit different as *Salinivenus iranica* reduced the percentage and size of colonies in all treated cells significantly in MDA-MB-231, MDA-MB-468, and MCF-7 cells, (*p* < 0.001). While *Salinibacter ruber* only declined colonies in MCF-7 cells (1.8-fold, *p* < 0.001) with no reduction in the size of colonies (Fig. 2a,7–12). The type of colonies also is a determinative factor for the presence of stem-like cells in a cell population as the higher number of holoclones (compact colonies with distinct margins) means that cancer stem cells were higher in the cell pools. The results exhibited that the number of holoclones reduced in *Salinivenus iranica* treated cells from 24.50 and 31.12 to 5.50 and 12.62 in MDA-MB-231 and MCF-7, respectively (*p* < 0.001) along with the reduction in meroclones (loose colonies) and paraclones (colonies with both compact and loose cells) in MDA-MB-468 cells. *Salinibacter ruber* only reduced the number of holoclones in MCF-7 cells from 31.12 to 16.75 (*p* < 0.001). However, it significantly enhanced the number of paraclones in MDA-MB-231 cells with no significant effect on the number of meroclones (Fig. 2b).

Our study found that treatment with *Salinibacter ruber* resulted in a significant decrease in the migration of breast cancer cells (*p* < 0.001). However, the invasion of MDA-MB-231 and MCF-7 cells was reduced by 60% (*p* < 0.001) and 77.5% (*p* < 0.001), respectively, while the invasion of MDA-MB-468 cells was increased by 89.1% (*p* < 0.001). Notably, the invasion index dramatically increased in cells treated with *Salinibacter ruber*, from 0.15 to 0.56 (*p* < 0.001) in MDA-MB-231 cells, from 0.04 to 0.60 (*p* < 0.001) in MDA-MB-468 cells, and from 0.74 to 6.00 (*p* < 0.001) in MCF-7 cells. Conversely, treatment with *Salinivenus iranica* had different effects on the invasion and migration of breast cancer cells. The migration of MDA-MB-231 and MDA-MB-468 cells increased by approximately two-fold (*p* < 0.001), while the migration of MCF-7 cells decreased by approximately 75% (*p* < 0.001). Specifically, *Salinivenus iranica* reduced the invasion of MDA-MB-231 and MCF-7 cells by 85.0% (*p* < 0.001) and 53.1% (*p* < 0.01), respectively, but did not significantly affect the invasion potential of MDA-MB-468 cells. Furthermore, *Salinivenus iranica* reduced the invasion index of MDA-MB-231 treated cells from 0.16 to 0.01 (*p* < 0.001). However, it significantly (*p* < 0.01) enhanced the invasion potential of MCF-7 by approximately two-fold, with no significant change in the invasion index of MDA-MB-468 cells (Fig. 2c). As the decrease in invasion index is an indicator of stem-like cells in cancer cell populations, and its reduction showed the reduction of cancer stem cells^14^, the results from *Salinivenus iranica* were better in reducing the cancer stem cells.

Overall, because of the more desirable results of *Salinivenus iranica* SM on inhibiting cancer stem cells (sphere and colony formation and invasion index), this strain was selected for future experiments.

### *Salinivenus iranica*’s supernatant metabolites induce late apoptosis and S phase inhibition in breast cancer cells

**Figure 3:**
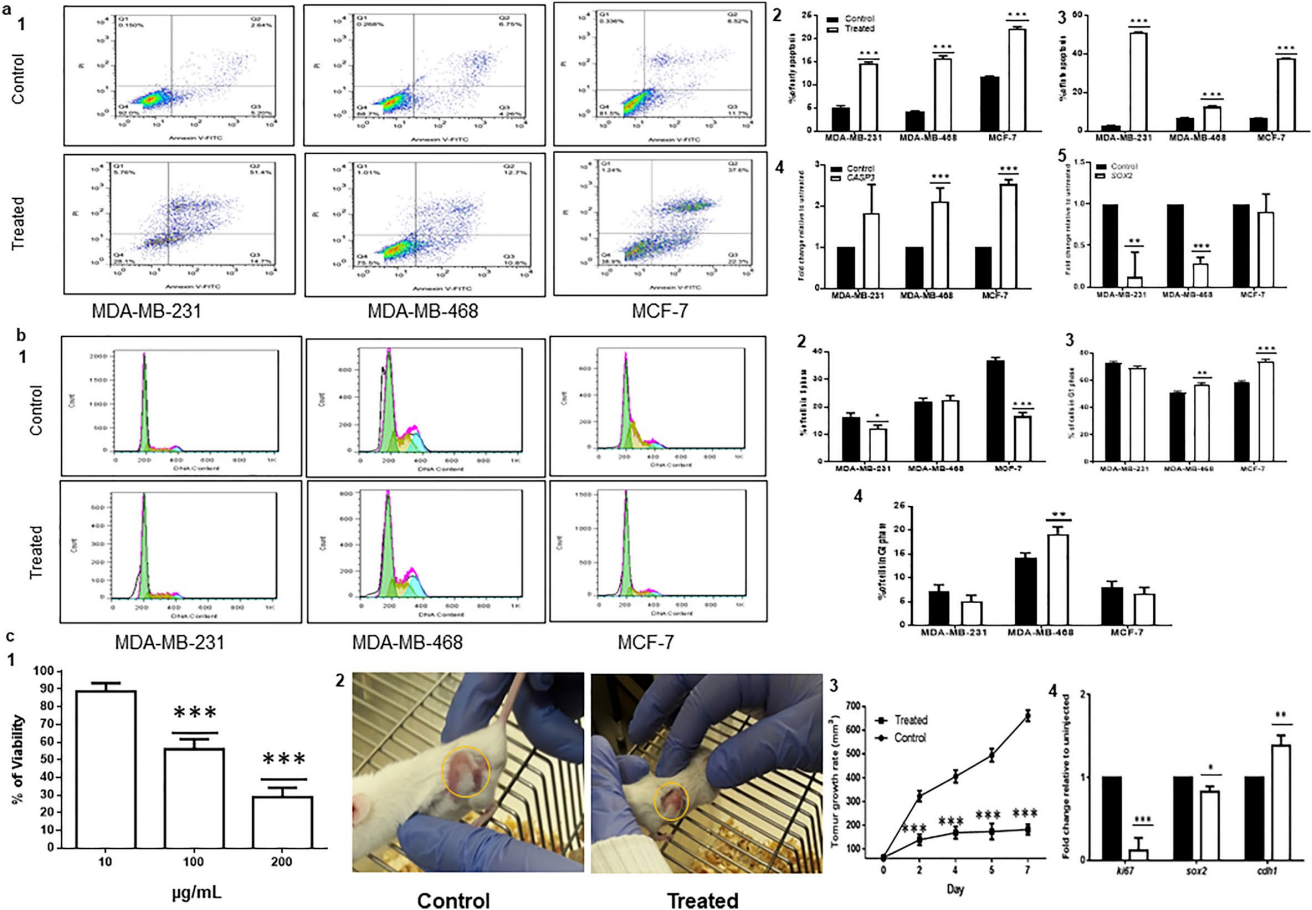
Effect of *S. iranica* SM on apoptosis and cell cycle of breast cancer cell lines and Tumorigenicity of the SM in breast cancer mice model. (**a**) Effects on apoptosis. 1. Apoptosis test of *Salinivenus iranica* SM on breast cancer cells. The number of cells in early (downright quadrate) and late (upright quadrate) apoptosis increased in treated cells in comparison with controls. 2. The percentage of early apoptosis. 3. The percentage of late apoptosis. 4. Quantitative real-time PCR analysis of *CASP3* in breast cancer cell lines. 5. Quantitative real-time PCR for *SOX2* (a pluripotency gene) in breast cancer cell lines. (**b**) Effects on cell cycle. 1. The cell cycle of *Salinivenus iranica* SM on breast cancer cells. 2. The number of cells in the S phase of the cell cycle after 48 h treatment with 100 µg/mL of *Salinivenus iranica.* 3. The number of cells in the G1 phase was up-regulated in MDA-MB-468 (*p* < 0.5) and MCF-7 (*p* < 0.001) significantly but was not changed on MDA-MB-231. 4. The number of cells in the G2 phase was up-regulated in MDA-MB-468 significantly (*p* < 0.05) but was not changed in MDA-MB-231 and MCF-7 cells. (**c**) Tumorigenicity in breast cancer mice model. 1. Viability of mouse breast cancer cell line (4T1) in presence of 10, 100, and 200 µg/mL of *Salinivenus iranica* SM. 2. Breast cancer mice models and their breast cell line tumors in control and treated (SM injected) groups. 3. Mean tumor volume of 4T1 cell line. 4. Quantitative real-time PCR for *ki67* (a marker of proliferation), *sox2* (a pluripotency gene) and *cdh1* (a migration-related gene) in 4T1 tumors. n = 4, *p < 0.05, **p < 0.01*,* ***p < 0.001.

The apoptosis assay was done on treated and untreated breast cancer cell lines to find the effect of *Salinivenus iranica* SM on the induction of apoptosis. As shown in Fig. 3a, the percentage of both early and late apoptosis was extremely enhanced in all treated cells, which were associated with overexpression of pro-apoptotic *caspase-3* (*CASP3*) (a marker of late apoptosis) (*p* < 0.001). Resistance to traditional anticancer therapy is related to *SOX2* expression. Therefore, inhibiting *SOX2* expression may decrease the malignant characteristics associated with breast cancer, such as invasion, migration, proliferation, stemness, and chemoresistance^15^. As a result, *SOX2* expression was monitored. Expression of the *SOX2* gene was significantly down-regulated in MDA-MB-231 and MDA-MB-468 cells (*p* < 0.001, Fig. 3a).

Cell cycle analysis showed that the percentage of cells in the S phase of the cell cycle significantly reduced in MDA-MB-231 (16.49% vs. 12.11%, *p* < 0.05) and MCF-7 cells (36.66% vs. 16.65%, *p* < 0.001) post-treatment, while no significant change was observed in MDA-MB-468. On the other hand, after treatment, the percentage of cells in the G1 and G2 phase was up-regulated significantly in MDA-MB-468 cells (50.95% vs. 56.45%, *p* < 0.01) and (14.10% vs. 19.16%, *p* < 0.01), respectively. Also, the percentage of cells in the G1 phase was up-regulated significantly in MCF-7 cells (58.21% vs. 74.18%, *p* < 0.001). In G1/G2 and G2 phase no significant changes were observed in MDA-MB-231 and MCF-7 cells, respectively (Fig. 3b).

### Salinivenus iranica inhibits breast tumor growth in vivo

We injected 1.25 × 10^6^ of 4T1 cells (mice breast cancer cell line) subcutaneously to develop breast cancer tumors in BALB/c mice. When the tumor size reached 50–60 mm^3^ (about three days post cellular injection), *Salinivenus iranica* SM at a dose of 17.5 mg/kg (Equivalent to 100 µg/mL in vitro) was injected intratumorally, just for one time. Interestingly, the size of injected tumors was smaller than controls post seven days of SM injection (181.60 mm^3^ vs. 661.74 mm^3^). The expression of *ki67* and *sox2* (markers of cell proliferation and pluripotency) was downregulated. However, *cdh1* (migration-related gene) was significantly upregulated in injected tumors (*p* < 0.01, Fig. 3c).

### A phenol Amine molecule acts as the effective part of *Salinivenus iranica* metabolites

**Figure 4:**
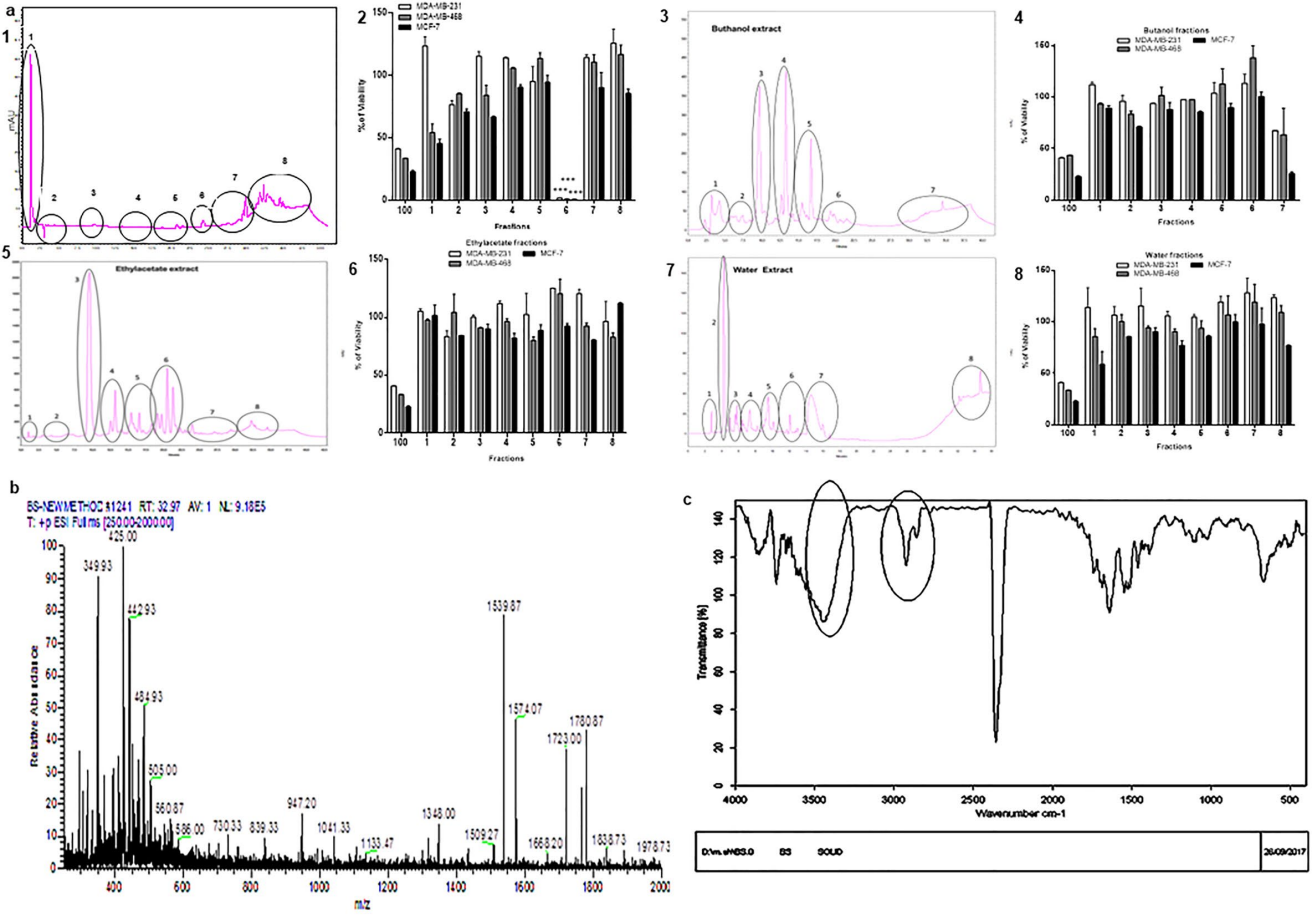
Characterization of the effective molecule of *Salinivenus iranica* Supernatant Metabolites. (**a**) Fractions evaluation. 1. Diagram of hexane fractions of *Salinivenus iranica* Supernatant Metabolites. The metabolites are divided into eight fractions. **2.** The viability of human breast cancer cell lines (MDA-MB-231, MDA-MB-468 and, MCF-7) in presence of 100 µg/mL of each hexane fraction of *Salinivenus iranica*. The 100 µg/mL of SM was used as the control (100 group). Fraction number six reduced the viability of all cell lines to 1%. 3. Diagram of butanol fractions of *Salinivenus iranica* Supernatant Metabolites. The metabolites are divided into seven fractions. 4. The viability of human breast cancer cell lines (MDA-MB-231, MDA-MB-468 and, MCF-7) in presence of 100 µg/mL of each butanol fraction of *Salinivenus iranica*. The fractions did not have any effect on the viability of cancer cell lines. 5. Diagram of ethyl acetate fractions of *Salinivenus iranica* Supernatant Metabolites. The metabolites are divided into eight fractions. 6. The viability of human breast cancer cell lines (MDA-MB-231, MDA-MB-468 and, MCF-7) in presence of 100 µg/mL of each ethyl acetate fraction of *Salinivenus iranica*. The fractions did not have any effect on the viability of cancer cell lines. 7. Diagram of water fractions of *Salinivenus iranica* Supernatant Metabolites. The metabolites are divided into eight fractions. 8. The viability of human breast cancer cell lines (MDA-MB-231, MDA-MB-468 and, MCF-7) in presence of 100 µg/mL of each water fraction of *Salinivenus iranica*. The fractions did not have any effect on the viability of cancer cell lines. (**b**) Mass spectrum of fraction number 6 of hexane factions from *Salinivenus iranica*. Based on this diagram the molecular weight of molecule without OH molecule is 1961.78 Da. (**c**) FT-IR diagram of fraction number 6 of hexane fractions from *Salinivenus iranica*. [M + NH4]^+^, [M + H]^+^, and [M + Na]^+^ ions were determined in this fraction. n = 4, ***p < 0.001.

To find the effective part of *Salinivenus iranica*'s SM, the crude metabolite was fractionated by four solvents: hexane, ethyl acetate, butanol, and water. It was separated into thirty-one fractions. A solution containing 100 µg/mL of separately fraction was prepared, and the viability of three human breast cancer cell lines was evaluated in their presence. Fraction number 6 of hexane had decreased the viability of all cell lines to 1% after 48 h, while the other 30 fractions did not exhibit any significant effect on cell lines viability (Fig. 4a). As the fraction had only one visible peak, the effective anticancer metabolite was purified in this phase. The mass spectrophotometry defined that this molecule's molecular weight (MW) was about 1961.78 Da (Fig. 4b). In Fourier Transform Infrared Spectroscopy (FT-IR) analysis, three types of functional groups were observed in this molecule (Fig. 4c). 1: In 2950 – 2850 cm^−1^, Alkyl C-H Stretch was distinguished, which are generally less useful in defining structure as a consequence of fairly ubiquitination. 2: In 3500—3300 cm^−1^, Amine N–H Stretch was detected, which is a primary amine, as Primary amines produce two N–H stretch absorptions, secondary amides only one, and tertiary none. 3: In 3550–3200 cm^−1^, Alcohol/Phenol O–H Stretch was detected. These results exhibited that the effective part of *Salinivenus iranica* metabolites was a phenol amine molecule with a molecular weight of 1961.78 Da.

**Figure 5:**
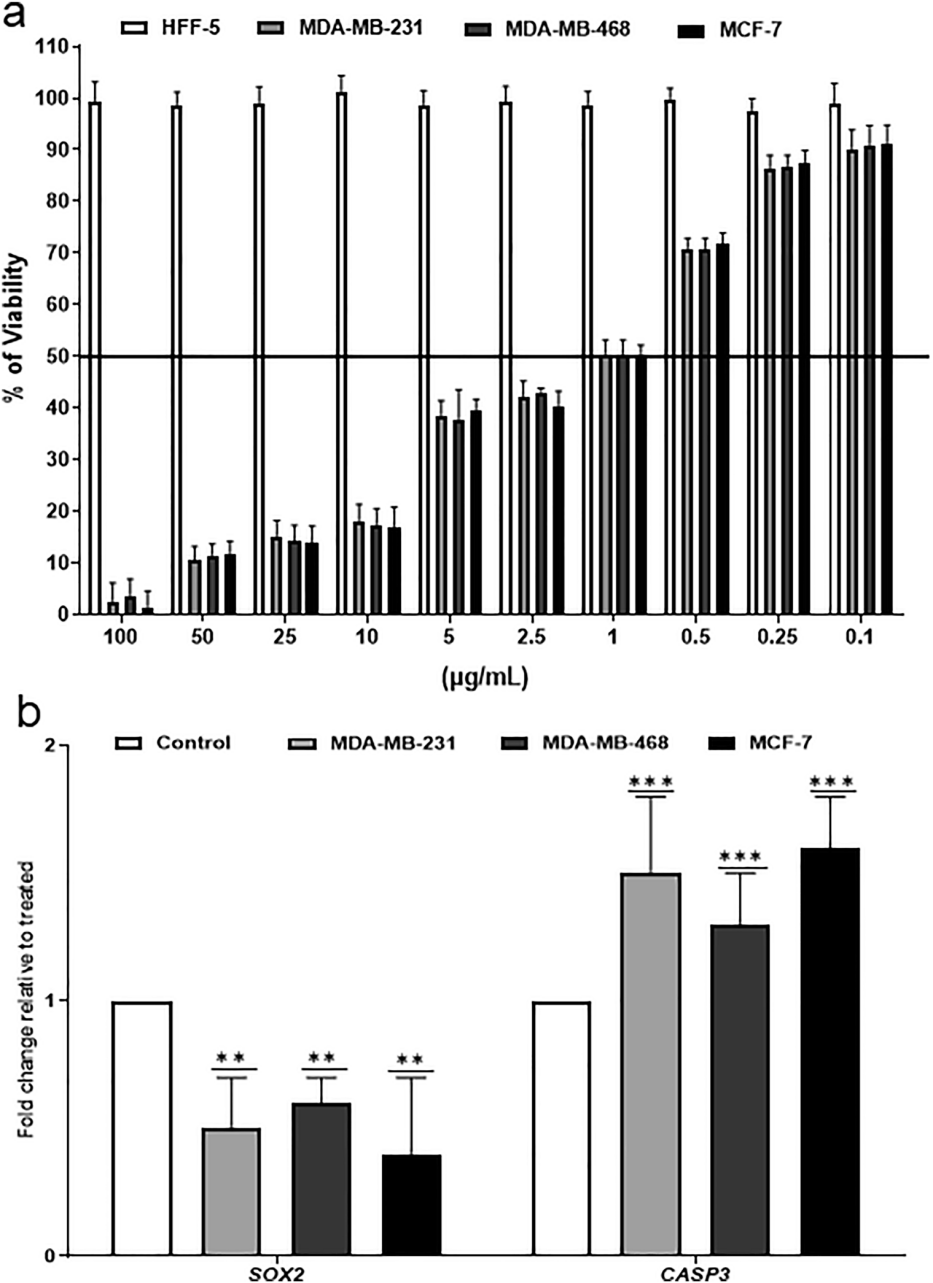
Anticancer activity of purified phenol amine molecule. (**a**) Finding the IC50 dose of phenol amine molecule on breast cancer cell lines (MDA-MB-231, MDA-MB-468 and, MCF-7) by MTT assay. The viability of cancer cells in 48 h reduced to 50% after treatment with a 1 µg/mL dose of phenol amine molecule. (**b**) Quantitative real-time PCR for *CASP3* (a marker of apoptosis), *SOX2* (a pluripotency gene) in breast cancer cell lines. Overexpression and downregulation of *CASP3* and *SOX2* were detected, respectively. n = 4, **p < 0.01, ***p < 0.001.

To determine the anticancer activity of purified phenol amine molecule, MDA-MB-231, MDA-MB-468, and MCF-7 breast cancer cell lines and one normal human fibroblast cell line (HFF-5) were subjected to treatment by different concentrations of this molecule. After 48 h, none of the concentrations had a cytotoxic effect on fibroblasts, while the 1 µg/mL of the phenol amine molecule significantly reduced the viability of all breast cancer cells to 50% (Fig. 5a). Also, this molecule significantly downregulated the expression of the pluripotency gene, *SOX2* (*p* < 0.001), and caused the overexpression of *CASP3* (a marker of late apoptosis) (*p* < 0.01, Fig. 5b) in all breast cancer cells.

## Discussion

Using natural compounds to target cancer stem cells could help decrease the severe side effects of chemotherapy and provide a potential anti-cancer response^8,10,16^. Therefore, in the present study, we screened supernatant metabolites (SM) of at least twenty-nine strains of halophilic bacteria isolated from different hypersaline lakes of Iran to find the effective anti-cancer strain that could potentially target most cancer cell lines, including breast (MDA-MB-468 and MCF-7), lung (A549) and prostate (DU145, PC3, and LNCaP) cancer cells. Our initial screening indicated that the SM of *Salinibacter ruber* and *Salinivenus iranica* reduced the viability of breast cancer cell lines at lower concentrations while it was ineffective on normal cells (HFF-5). At higher concentrations, these bacteria were able to decrease the viability of all selected cancer cell lines. This suggested that their effects might be dose-dependent. SMs enhanced the percentage of early and late apoptosis and the expression level of *CASP3* in breast cancer cells. Sagar et al. observed that halophilic bacterial extracts were toxic at the dose of 200 and/or 500 µg/mL on HeLa, MCF-7, and DU145 cells^17^ and induced apoptosis in cancer cells^18^. Apoptotic cell death is induced by caspase-3, which is an executioner caspase and a crucial mediator of intrinsic and extrinsic pathway-activated apoptosis. Upon activation of pro-apoptotic proteins, caspase-3 cleaves proteins such as cell cycle proteins, and DNase proteins and leads to apoptotic cell death^19^. More so, caspase-3, recognized as an important early biomarker for evaluating chemotherapy-induced cell death, is mainly responsible for tumor cells' response to chemotherapy via the cell apoptosis pathway and activation^20^. Furthermore, the molecule, PPDHMP, from *Staphylococcus* sp. strain MB30 (a marine bacterium) induces apoptosis in the lung (A549) and cervical (HeLa) cancer cells through increases in the expression of *CASP3*^21^. Moreover, the number of cells in the cell cycle's S phase (synthesis phase) significantly reduced in MDA-MB-231 and MCF-7 cells post-treatment with *Salinivenus iranica*. Therefore, *Salinivenus iranica* could inhibit the synthesis phase in breast cancer cells.

Interestingly, *Salinivenus iranica* SMs significantly reduced the number and size of spheres and colonies in all breast cancer cell lines, which were associated with the downregulation of *SOX2* as a pluripotency gene. *SOX2* is an essential gene in breast cancer cells that plays an early role in breast carcinogenesis and induces the metastatic potential in these cells^22^, and its inhibition could prevent tumor initiation of human breast cancer^23^. In contrast, *Salinibacter ruber* SMs potentially reduced the number and size of spheres and had no reduction effect on colony formation ability of all breast cancer cell lines. Both sphere and colony formation can indicate the presence of cancer stem cells in breast cancer cell lines^12,24^. More so, holoclones, which are compact colonies with specific borders, are an important marker of cancer stem cells^24^. *Salinivenus* *iranica* reduces the number of holoclones in MDA-MB-231 and MCF-7 cells. In contrast, the *Salinibacter* *ruber* reduced these numbers only in MCF-7 and enhanced the number of paraclones in MDA-MB-231, which is a pattern of induction of differentiation. Moreover, cancer stem cells are assumed as metastasis inducers in breast cancer^16^, and the invasion index is an exhibitor of invasion in cancer cells. Our results indicated that the *Salinibacter* *ruber* significantly increased this index in all breast cancer cells, but *Salinivenus* *iranica* reduced this index in MDA-MB-231 cells. Therefore, we suggested that *Salinivenus* *iranica* is a more reliable strain for inhibiting cancer stem cells. In vivo examinations showed that the tumor size decreased in the presence of *Salinivenus* *iranica* SM. Halophilic bacterial extracts exhibited tumor growth inhibition ability in previous studies. It was reported that *Streptomyces* sp. ANAM-5 and AIAH-10 in their co-culture produced some metabolites which had decreased tumor growth in Ehrlich Ascites Carcinoma cell-bearing albino mice^25^. The expression of *ki67* was significantly down-regulated in treated tumors compared to the untreated ones. As *ki67* is the marker of proliferation^26^, this revealed the role of the SM in the proliferation inhibition of breast cancer cells. Also, the expression of *sox2* (a pluripotency gene) was reduced in treated tumors, which approved the in vitro studies. The upregulation of gene *cdh1*, which encodes E-cadherin, is important in inhibiting metastasis in the breast cancer tumor cells because the inhibition of *cdh1* causes the initiation of epithelial-mesenchymal transition and metastasis^27^. The expression of *cdh1* was enhanced in mouse breast cancer tumors after *Salinivenus iranica* treatment, and it was similar to the effect of Salinomycin on mouse breast cancer stem cells^28^. This showed the similarities between Salinomycin and *Salinivenus iranica* SM in inhibiting breast cancer stem cells. Moreover, the purified phenol amine molecule reduced the viability of breast cancer cells at a lower concentration with a significant decrease in *SOX*-2 expression. This revealed that the purified phenol amine was the effective molecule that performed the principal role in inhibiting breast cancer stem cells in *Salinivenus iranica* SM.

## Conclusions

This is the first study to investigate the anti-cancer stem cell effects of halophilic bacterial metabolites. The findings revealed that metabolites produced from *Salinivenus iranica* have significant anti-cancer and anti-cancer stem cell activities. Furthermore, the anti-cancer stem cell ability of the purified phenol amine molecule was discovered in this study. The apoptosis was induced by overexpression of *CASP3*, and the pluripotency was reduced by the downregulation of *SOX2*. In addition, the SM decreased the invasion index of breast cancer cell lines and demonstrated these results in tumors of the mouse model.

## Methods

### Bacterial culture, supernatant isolation, and metabolite extraction

Twenty-nine halophilic strains were selected from the Iranian Biological Resources Center (IBRC) microorganism’s bank. All strains cultured in Sea Water Nutrient (SWN) medium with the total salt of 3% or 7% or in Modified Growth Medium (MGM) with 23% total salt or Yeast Malt Agar (YM Agar or ISPII medium, Table 1). All the materials and reagents were purchased from Merck (E. Merck, Darmstadt, Germany). One loopful of agar slant culture of each strain for metabolite production was inoculated into 25 mL of proper medium. The inoculated medium was incubated at 34 or 40 °C for 12 h on a rotary shaker at 150 rpm and then transferred into a 225 mL medium to cultivate at the same temperature for 3 or 7 days. The culture was taken and centrifuged at 4000 g for 40 min. The cell-free supernatant was filtered with Whatman no.1 filter paper. An equal volume of ethyl acetate was added to the upper layer with the ratio of 1:1 and was shaken well for 2 h at room temperature, and was allowed to settle overnight at 4 °C. The upper organic layer containing supernatant metabolites was collected in a clean, dry bottle and then evaporated by BUCHI Rotavapor R_114 (Switzerland) until 1 mL of total volume remained in the rotary balloon. The remaining volume was transferred to a sterile vial with identified weight and evaporated again to dry completely. The vial which contained the dried metabolite was weighted, and the pure weight of the metabolite was calculated. The SM was dissolved in DMSO (Merck, Germany), serving as a stock solution, which was later diluted to a final solvent concentration. The SM was tested as the weight of total SM/volume^9^. The SM of each inoculated medium was isolated and used as a control negative.

### Human cell culture

HFF-5 (Human Foreskin Fibroblast) cells were obtained from the Royan Institute cell bank (Tehran, Iran). Prostate cancer cell lines of PC3, LNCaP, and DU145 were obtained from the national cell bank of Iran (Pasteur Institute of Iran, Tehran, Iran). Lung cancer cell line, A549 (IBRC C10080) and breast cancer cell lines, MDA-MB-468 (IBRC C 10095), MDA-MB-231(IBRC C10684), MCF-7 (IBRC C10082), and 4T1 (IBRC C10056) were obtained from the cell bank of Iranian Biological Resource Center, Tehran, Iran. The monolayer of Breast cancer cell lines was grown in DMEM high glucose (GIBCO; Germany) medium, and other cell lines were grown as a monolayer in RPMI 1640 (GIBCO; Germany) medium. All cells were grown in media supplemented with 10% (v/v) Fetal Bovine Serum (FBS), 1.0 mM sodium pyruvate and 2 mM L-glutamine, 100 U/mL penicillin, and 100 μg/mL streptomycin (all from GIBCO; Germany) at 37 °C in a humidified atmosphere of 95% air and 5% CO_2_^29^.

### MTT assay

The effect of SMs on cell viability was evaluated using the MTT-assay by 3-(4,5-Dimethylthiazol-2-yl)-2,5-diphenyl tetrazolium bromide (MTT, Biosera, Austria), and the results were expressed as viable cell percentages compared to the control. A 96-well plate was seeded with approximately 5000 cells per well and allowed to adhere for 24 h. Three replicates of each plate were incubated with different concentrations of metabolite extracts (0, 10, 100, 200, 400, and 800 µg/mL) or purified molecules (100, 50, 25, 10, 5, 2.5, 1, 0.5, 0.25, and 0.1 µg/mL), and viability was measured after 48 h^30^. The final concentration of DMSO was 1mM which was not cytotoxic for our cell lines (data have not been shown). After treatment, 10 μL of 5 mg/mL solution of MTT in PBS was added to each well. The plate was then incubated for 3 h at 37 °C. Finally, the medium was removed, and 100 μL of the DMSO was added to each well to solubilize the blue formazan. Dye absorbance was measured at 560 nm. One chamber of each 96 well plate contained only DMSO, and its OD_560_ was assumed blank. The resulting ODs (control and treated groups) from each plate were first subtracted from the blank of the same plate, and then the mean of OD_560_ for control and treated groups was calculated. The percentage of viable cells was calculated via mean OD_560_ (Treated group)/ mean OD_560_ (Control group) × 100. The effect of SM from inoculated mediums was also measured in the same way, and no significant changes were observed (data have not been shown).

### Sphere formation assay

Sphere formation capacities were assessed after 48 h of SM pretreatment. To perform this assessment, cells were seeded at a density of 10^6^ cells/well in 6-well plates and allowed to adhere for 24 h. Then the cells were treated with bacterial metabolite at 0 (as control) and 100 µg/mL (IC50 dose). After 48 h, cells were harvested for sphere formation assay. To create a single-cell suspension for sphere formation, the single-cell suspension was prepared by enzymatic (1 × Trypsin–EDTA, Sigma Aldrich) and manual disaggregation (25-gauge needle). Cells were seeded in culture dishes coated with (2-hydroxyethyl methacrylate) (poly-HEMA, Sigma) and cultivated in serum-free DMEM supplemented with B27 (GIBCO, Karlsruhe, Germany), 20 ng/ml EGF and bFGF (Royan Institute, Tehran, Iran), and PenStrep. Cells were grown for 7 days and maintained in a humidified incubator at 37 °C and an atmospheric pressure of 5% (v/v) carbon dioxide/air. After 7 days, the spheres with a diameter of > 50 µm were counted using an eyepiece graticule. The percentage of spheres pre-and post-treatment was calculated by dividing the number of spheroids by the number of seeding number × 100 and is referred to as the percentage of sphere formation. Values were expressed as means ± SD of at least three independent experiments.

### Colony formation assay

Colony formation capacities were assessed after 48 h of SM pretreatment. The numbers of 200 cells from pretreated and un-pretreated (Control) groups were plated in each well of six-well plates in DMEM medium supplemented with 10% (v/v) FBS and allowed to grow for 12 days. After this, the upper media was removed, and the chamber was washed twice with PBS. Then the colonies were fixed with paraformaldehyde 4% and stained with crystal violet. The colonies were counted under light microscopy, and the percentage of colonies pre-and post-treatment was calculated by dividing the number of colonies by the number of seeding number × 100, which is referred to as the percentage of colony formation. Values were expressed as means ± SD of at least three independent experiments.

### Invasion and migration assay

Invasion and migration assays were done after 48 h of SM pretreatment. To assess migration, 125 × 10^3^ were seeded in the serum-free medium into the upper chamber of the transwell filter (pore size 8 µm, Corning; Germany). The bottom of the wells (24-well plate) was filled with DMEM medium supplemented with 20% (v/v) FBS. To assess invasion, the same procedure was done with only one difference: Transwell filters were coated with 0.5 mg/mL of Matrigel overnight later, before seeding. For both migration and invasion assay, the media and remaining cells in the upper chamber were removed 12 h after seeding, and the lower side of the filters was washed twice with PBS and fixed with paraformaldehyde 4%, then they were colored with crystal violet. The number of migrated and invaded cells was counted and compared between treated and untreated (Control) cells. The invasion index was calculated as the number of invaded cells/numbers of migrated cells.

### Quantitative real-time RT-PCR (qRT-PCR)

Total mRNA was isolated from treated and control cell lines after 48 h using TRIzol reagent (Invitrogen) according to the manufacturer’s instruction. cDNA was synthesized using the RevertAid H Minus First Strand cDNA Synthesis Kit (Fermentas, Waltham, Massachusetts, USA). SYBR Premix Ex Taq II (Tli RNase H Plus) (Takara, Berkeley, California, USA) was utilized to perform quantitative real-time reverse transcription-PCR (RT-PCR) using Rotor-Gene 6000 (Corbett). The *GAPDH* and *gapdh* gene transcripts were measured as a normalizer to determine the other gene relative transcripts (2^−ΔΔCt^)^30^. The sequences of primers are *GAPDH:* 5′ GTG GTC TCC TCT GAC TTC AAC 3′, 5′ AGG GTC TCT CTC TTC CTC TTG 3′, *SOX2:* 5′ CAA GAT GGC CCA GGA GAA C 3′, 5′ TCA TGT AGG TCT GCG AGC TG 3′, *CASP3:* 5′ AAG CGA ATC AAT GGA CTC TGG 3′, 5′ CAA GTT TCT GAA TGT TTC CCT GAG 3′, *gapdh:* 5′ CAA GGA GTA AGA AAC CCTG 3′, 5′ TCT GGG ATG GAA ATT GTG AG 3′, *ki67:* 5′ AGA GCC TTA GCA ATA GCA ACG 3′, 5′ GTC TCC CGC GAT TCC TCT G 3′, *sox2:* 5′ GCT GGG AGA AAG AAG AGG AG 3′, 5′ ATC TGG CGG AGA ATA GTT GG 3′, *cdh1:* 5′ TCG GAA GAC TCC CGA TTC AAA 3′, 5′ CGG ACG AGG AAA CTG GTC TC 3′.

### Apoptosis test

The apoptotic effects of SM on treated and control cells were measured using the annexin-V/PI Staining Kit (Sigma) according to the manufacturer’s instruction. Annexin-V binding to phosphatidylserine suggests early apoptosis, whereas binding to annexin-V and PI indicates late apoptosis^31^.

### Cell cycle test

Cell cycle analysis was measured by flow cytometry. After 48 h of treatment, treated and untreated (control) cells were fixed in cold 70% alcohol at 4 °C overnight. The fixed cells were then treated with 0.25% Triton X-100 for 5 min in an ice bath and stained in propidium iodide (PI) solution (50 μg/mL, Sigma; USA) which contained 0.1 mg/mL RNase for 1 h in a dark room at 37 °C. Cell cycle analysis used by FACScan flow cytometer (BD Biosciences, Bedford, MA).

### Mouse tumorigenicity assay

The suspension of 1.2 × 10^6^ cells (4T1 cells) was prepared in 20 µL of Matrigel (0.34 mg/mL, BD Biosciences, San Jose, CA, USA). Six BALB/c female mice were aged 4–6 weeks (were purchased from Pasteur Institute of Iran and maintained in a clean area). Animals were allowed to acclimate for two weeks prior to experimentation. All mice were injected with the 1.2 × 10^6^ cells subcutaneously, with one injection on the flank of each animal. Once tumors got palpable (about 4 days post-injection), tumor volumes were calculated using the formula: (L × W^2^)/2 which L and W indicated length and width, respectively. The length and width were measured with a caliper 2,4,5 and 7 days after intratumor metabolite injection^30^. To find the proper dose of SM intra-tumor injection, we considered the 100 µg/mL dose of in vitro injection as 100 mg/kg^32,33^. Because the minimum weight of our mice was 17.5 g, we injected 1.75 mg of SM intratumorally in each mouse in the treated group. We select the minimum dose to avoid the death of mice. The SM was dissolved in DMSO (total volume 70 µL), and then an intra-tumor injection was done. Three tumors were injected with only DMSO (70 µL) as the control. Mice were sacrificed in a humane manner when the tumor size exceeds 25 mm in diameter in either direction. This study was carried out in accordance with the recommendations of the animal care committee of the Chancellor for Royan Institute. The protocol was approved by the animal care committee of Royan Institute (Tehran, Iran, approval number 931119) and was performed in accordance with the ethical standards. All authors complied with the ARRIVE guidelines. Human data are not involved in this study.

### High-performance liquid chromatography

The chromatography experiments were performed by Knauer HPLC pump model S 1000 and controller, autosampler model S 3900, and photodiode array detector model S 2800. The Eurospher C18 column 250 × 4.6 mm with precolumn, 100 A°- 5µm, was used. The Bacterial SM was analyzed by gradient method using water/methanol as a mobile phase with the following gradient program: 0 min- 90% water, 5 min- 57% water, 11 min- 30% water, 16 min- 0% water, 21 min- 0% water, 26 min- 90 water. The flow rate was 1 mL/min, and the injection volume was 100 μL.

### Mass spectrometry

The ESI–MS/MS system, ThermoFisher Scientific (Bremen, Germany) ion trap mass spectrometer (model LCQ, mass range m/z 10–2000), was used to identify fraction number 6. The Xcalibur software was used to conduct instrument control, data acquisition, and processing. Typical positive ESI–MS conditions were: capillary voltage + 46 kV; spray voltage + 5 kV; tube lens: − 60 kV and capillary temperature: 300 °C.

### Fourier transform infrared spectroscopy (FT-IR)

To identify the functional groups of extracted compounds, IR spectra were recorded at 5 cm^−1^ resolutions with 60 scans using Bruker Tensor 27 spectrometer (USA).

### Statistical analysis

The one-way ANOVA test was used to determine the statistical significance of the differences between the treated and the control groups. All of the experiments were performed in triplicates or more, and the data obtained were expressed as means ± SD. *p* < 0.05 were considered statistically significant.

## Data Availability

All data generated or analysed during this study are included in this published article.
